# Raghu Gaind, FRCP, FRCPsych, DPM

**DOI:** 10.1192/bjb.2022.56

**Published:** 2023-04

**Authors:** Steven Hirsch

Formerly Physician in Psychological Medicine, Guy's Hospital, London, UK

Raghu Gaind, who died on 7 September 2021 aged 86, was a man of immense energy, and had an extraordinarily diverse career. After working as an NHS consultant in psychiatry, he took early retirement at 50 and then ran a large private practice in London. He set up two private nursing homes (Suttons Manor psychiatric clinic in Romford, Essex, and a clinic for the elderly mentally infirm in Beckenham, Kent). He organised numerous successful courses for psychiatrists in the UK and abroad. He served as an examiner in psychiatry in numerous countries throughout the world. For 30 years he edited a series of books titled *Current Themes in Psychiatry*, which over five editions summarised contemporary thinking on the subject. He was active in social psychiatry, editing the *International Journal of Social Psychiatry* for 5 years. For some years he was chair of the now closed Institute of Social Psychiatry, and was elected Secretary General of the World Association for Social Psychiatry in 1985, serving in this capacity until 1991.

Most notable, however, were his philanthropic activities, undertaken particularly in India. He was Chairman of the Arpana Charitable Trust (UK), which helped finance a 125-bed hospital in Chandigarh, India, with community outreach services concentrating on the empowerment of women in rural communities among the poorest of the poor. With Prince Charles (now King Charles III) as its royal patron, this charity has raised funds totalling around £4.5 million over 20 years. It has carried out pioneering work in Delhi, where it was involved in the resettlement of some 30 000 street dwellers and inhabitants of shanty towns. Raghu was patron of a number of other Indian charities, including the Maitreey Mission, which is involved in the treatment and rehabilitation of people with leprosy living in colonies in and around Delhi. He also helped to create facilities for young children from a leprosy colony (Ramakrishna Puram, in Delhi). In 1992 he was awarded the title Distinguished Citizen of India by the Indian Government for his social contributions. In later years he publicised the plight of Tibetan refugees, particularly those in the Chamba District.

Raghu was born in Jammu, Kashmir, to Meher Chand Gaind, a barrister and Gian Devi. His family had served the maharajas for five generations, one member becoming Finance Minister to the state of Kashmir. As a child he learned Urdu and Farsi. He qualified from Amritsar Medical College in 1954 and shortly thereafter came to England to train in neurology at the National Hospital, Queen Square, and in psychiatry at the Maudsley Hospital and Institute of Psychiatry, London. While training and in his first years as a consultant, to support his large family (eventually he had six children), his ability for creative and prodigious work was exemplified by the fact that he worked as a general practitioner at weekends and as a police surgeon. He was regularly away from home from 8 a.m. to midnight. On one occasion he saw the same patient as a police surgeon in the emergency clinic at the Maudsley and as a registrar on the hospital ward. He was also actively involved at that time in what turned out to be highly influential research into drug medication in chronic schizophrenia.^1^ He was appointed Physician in Psychological Medicine to Guy's Hospital in 1969 and Chairman of the Psychiatry Department in 1973, with beds in what was then St Olaves, Bermondsey and Bexley Hospitals.

His strong connection with the Middle East began in 1970, when he went with three other consultants to Saudi Arabia to help commission the King Faisal Specialist Hospital in Riyadh. He learned Arabic while he worked there for four and a half months. Subsequently, once a month he travelled to Saudi Arabia for a busy weekend seeing private patients. From 1970 to 1981 he was advisor in mental health to the Kingdom of Saudi Arabia. His global commitments rapidly extended. He was visiting professor and examiner to the University of Malta in 1980, the West Indies in 1981–1984, the University of California, Los Angeles (UCLA) in 1984–1985, the Punjab in 2003–2005 and Nairobi, Kenya, in 2007. He was awarded an Emeritus Professorship in Neuropsychiatry at Guru Ram Das College Amritsar, Punjab, in 2002.

As a result of his extraordinary capacity for hard work, his amazing charm and his ability to make warm, lasting friendships, combined with his considerable creative intelligence, Raghu did much to educate and entertain his colleagues. In 2011 he self-published a 700-page autobiography,^2^ which makes interesting and amusing reading.

In 1959, he married June Beddoe, with whom he had six children. They divorced in 1987, and in 1989 he married Dr Susan Davenport, a consultant psychiatrist. She died from cancer after a short illness in August 2020. In 2000 Raghu self-diagnosed Parkinsonism and battled its slow progression over 20 years in a determined and courageous manner, meeting friends, playing bridge, going on cruises and travelling with Susan. He is survived by his first wife, his children Anil, Sushilla, Gita, Tripta, Shoba and Ranjit and their respective spouses, 15 grandchildren and 4 great grandchildren.
